# Supplementary material for: Association of women-specific health factors in the severity of Parkinson’s disease
Source: NPJ Parkinsons Dis. 2023 Jun 5;9:86. doi: 10.1038/s41531-023-00524-x (PMC10241917; doi:10.1038/s41531-023-00524-x)
Supplement: Supplementary file 1 — Supplementary Material [file 41531_2023_524_MOESM1_ESM.pdf]

Supplemental Table 1A. Part I: Univariate Logistic Regression Results

	Sample Size	OR (95% CI)	p-value
**Age**			
	160	1.01 (0.97, 1.05)	0.6
Hispanic			
	160	0.32 (0.01, 10.05)	0.52
**White**			
	160	2.20 (0.25, 19.32)	0.48
**Genetic Status**	158		
GBA		0.31 (0.05, 1.83)	0.2
LRRK2		0.63 (0.11, 3.71)	0.61
GBA	160	0.69 (0.21, 2.24)	0.54
LRRK2	160	1.51 (0.47, 4.88)	0.49
PRKN	160	5.00 (0.88, 28.29)	0.069
None		0.41 (0.10, 1.74)	0.23
Other		-reference-	
**Medications**			
Levodopa	160	1.66 (0.74, 3.70)	0.22
Entacapone	159	17.14 (0.55, 533.12)	0.11
Carbidopa, Levodopa, and Entacapone (Stalevo)	158	2.56 (0.78, 8.40)	0.12
Pramipexole	158	1.42 (0.55, 3.66)	0.47
Ropinirole	158	4.68 (1.30, 16.85)	***0.018***
Rotigotine	158	1.19 (0.21, 6.72)	0.84
Rasagiline	158	0.71 (0.29, 1.70)	0.44
Selegiline (oral, sublingual)	159	1.16 (0.10, 13.10)	0.9
Amantadine (liquid, infusion)	158	0.47 (0.13, 1.73)	0.26
**Current Comorbidities**			
Arrythmia/Atrial Fibrillation	160	1.00 (0.25, 4.04)	0.99

Arthritis	160	1.96 (0.97, 3.94)	0.059
B12 deficiency	160	1.95 (0.68, 5.60)	0.21
Cancer	160	1.17 (0.21, 6.64)	0.86
Depression	160	6.17 (2.89, 13.19)	***<0.001***
Diabetes Mellitus (adult onset)	160	2.42 (0.47, 12.45)	0.29
Hearing Loss	160	1.94 (0.91, 4.12)	0.087
Hyper cholesterolemia (High Cholesterol)	160	1.50 (0.72, 3.13)	0.28
Hypertension (High Blood Pressure)	160	0.82 (0.34, 1.99)	0.66
Loss of smell	160	0.84 (0.42, 1.68)	0.63
Lung disease (including emphysema)	160	2.05 (0.60, 7.09)	0.25
Other	160	2.11 (1.06, 4.21)	***0.034***
Peripheral neuropathy	160	0.69 (0.21, 2.24)	0.54
PTSD	160	5.19 (1.24, 21.71)	***0.024***
Recreational drug use	160	1.18 (0.28, 4.92)	0.82
Renal insufficiency (kidney disease)	160	2.39 (0.33, 17.49)	0.39
Thyroid disease (not cancer, including Grave's disease)	160	1.53 (0.68, 3.46)	0.3
**Still Menstruating Variable**			
	160	1.07 (0.35, 3.26)	0.91
**Diagnosis of PMS Variable**			
	160	1.86 (0.61, 5.68)	0.28
**Currently Perimenopausal Variable**			
	156	0.96 (0.24, 3.89)	0.96
Hormone Levels Checked for Perimenopausal Variable			
	10	15.40 (0.41, 584.08)	0.14
**Have Experienced Menopause Variable**			
	157	1.21 (0.44, 3.28)	0.71
**Menses and PD Medication**			
Medication felt less effective	160	10.09 (1.10, 92.81)	***0.041***

More off/irregular frequency periods	160	5.00 (0.88, 28.29)	0.069
**Hormone Replacement Therapy Variable**			
	152	1.48 (0.69, 3.15)	0.31
**Birth Control Variable**			
	110	0.68 (0.07, 6.74)	0.74
**Experience of Breast Feeding**	159		
Yes		1.62 (0.64, 4.12)	0.31
No		1.60 (0.47, 5.47)	0.45
N/A		-reference-	
**PD Onset**	157		
While I was still having regular periods		-reference-	
While I was going through perimenopause		1.58 (0.42, 5.94)	0.5
One year or more after my last menstrual period		1.00 (0.40, 2.50)	0.99
**History of Surgeries**			
Total hysterectomy (removal of uterus, cervix, ovaries, Fallopian tubes, and surrounding structures)	160	1.58 (0.66, 3.79)	0.31
Oophorectomy (surgical removal of one/unilateral or both/bilateral ovaries)	160	1.86 (0.61, 5.68)	0.28
Mastectomy	160	0.46 (0.05, 4.00)	0.48
Partial hysterectomy (just uterus)	160	1.51 (0.47, 4.88)	0.49
**Types of Hormone Replacement Therapy**			
Estrogen	160	1.74 (0.62, 4.89)	0.29
Progesterone/Progestin	160	1.58 (0.26, 9.77)	0.62
Estrogen and progesterone combination	160	1.00 (0.36, 2.78)	0.99
**Types of Birth Control**			
Pill	160	1.66 (0.76, 3.62)	0.2
Ring	160	0.57 (0.06, 5.28)	0.62
Hormonal IUD	160	0.76 (0.20, 2.95)	0.7
Copper IUD	160	1.46 (0.50, 4.27)	0.49

Tubal ligation	160	4.33 (1.64, 11.45)	***0.003***
**Pregnancy Experiences**			
In vitro fertilization	160	0.25 (0.01, 6.63)	0.41
C-section	160	0.55 (0.21, 1.46)	0.23
Vaginal birth	160	3.48 (1.49, 8.13)	***0.004***
Having children prior to PD diagnosis	160	3.44 (1.25, 9.47)	***0.017***
No children	160	0.35 (0.11, 1.08)	0.068
**Difficulties in Pregnancy Experiences**			
Conceiving (fertility)	160	1.76 (0.70, 4.44)	0.23
Childbirth	160	3.03 (1.19, 7.73)	***0.02***
Pregnancy (reaching full birth)	160	1.40 (0.55, 3.60)	0.48
**Pregnancy-related Aliments**			
Eclampsia or pre-eclampsia	160	0.57 (0.12, 2.77)	0.48
Diabetes during pregnancy	160	28.47 (1.17, 692.41)	***0.04***
Viral infection	160	7.02 (0.08, 647.52)	0.4
Bacterial infection	160	5.00 (0.88, 28.29)	0.069
Depression/anxiety during pregnancy	160	22.20 (2.69, 183.12)	***0.004***
Postpartum Depression	160	6.36 (2.07, 19.52)	***0.001***
Flair up migraines	160	3.02 (0.96, 9.51)	0.06
**Hormone-related Disorders**			
Polycystic Ovarian Syndrome	160	3.30 (0.71, 15.37)	0.13
Hypothyroidism	160	1.12 (0.45, 2.80)	0.81
Hyperthyroidism	160	0.57 (0.12, 2.77)	0.48
Diabetes Type II	160	0.93 (0.17, 4.97)	0.93
Migraines (no aura)	160	2.12 (0.82, 5.51)	0.12

Variables in italics: use Firth method
Dummy variables for each response option
Reference was used to for univariate modeling and for the outcome of moderate/severe phenotype

Supplemental Table 1B. Part II: Univariate Logistic Regression Results

	Sample Size	OR (95% CI)	p-value
**Age**			
	302	1.02 (0.98, 1.05)	0.33
**Hispanic**			
	300	0.72 (0.09, 6.11)	0.76
White			
	299	6.62 (0.35, 125.61)	0.21
**Genetic Status**	299		
GBA		1.07 (0.26, 4.31)	0.93
LRRK2		0.50 (0.10, 2.58)	0.41
GBA	302	1.39 (0.56, 3.41)	0.48
LRRK2	302	0.61 (0.17, 2.11)	0.43
PRKN	302	1.68 (0.43, 6.56)	0.45
None		0.80 (0.25, 2.54)	0.7
Other		-reference-	
**Medications**			
Levodopa	302	2.20 (1.03, 4.73)	***0.043***
Entacapone	300	1.77 (0.33, 9.37)	0.5
Carbidopa, Levodopa, and Entacapone (Stalevo)	299	1.96 (0.72, 5.36)	0.19
Pramipexole	299	1.30 (0.53, 3.20)	0.56
Ropinirole	299	2.72 (1.13, 6.52)	***0.025***
Rotigotine	299	3.31 (1.01, 10.83)	***0.048***
Rasagiline	299	0.52 (0.23, 1.16)	0.11
Selegiline (oral, sublingual)	300	0.30 (0.04, 2.32)	0.25
Amantadine (liquid, infusion)	299	2.09 (0.99, 4.43)	0.053
**Current Comorbidities**			
Arrythmia/Atrial Fibrillation	302	1.24 (0.44, 3.50)	0.68
Arthritis	302	1.06 (0.59, 1.89)	0.85

B12 deficiency	302	1.00 (0.36, 2.76)	0.99
Cancer	302	1.62 (0.56, 4.71)	0.37
Depression	302	2.94 (1.61, 5.38)	***<0.001***
Diabetes Mellitus (adult onset)	302	0.87 (0.19, 4.10)	0.86
Hearing Loss	302	1.76 (0.95, 3.27)	0.074
Hyper cholesterolemia (High Cholesterol)	302	0.63 (0.32, 1.27)	0.2
Hypertension (High Blood Pressure)	302	1.27 (0.65, 2.47)	0.48
Loss of smell	302	1.93 (1.08, 3.47)	***0.027***
Lung disease (including emphysema)	302	1.51 (0.53, 4.34)	0.44
Other	302	0.97 (0.53, 1.79)	0.93
Peripheral neuropathy	302	2.93 (1.38, 6.23)	***0.005***
PTSD	302	1.50 (0.47, 4.84)	0.5
Recreational drug use	302	0.66 (0.15, 3.03)	0.6
Renal insufficiency (kidney disease)	302	3.43 (0.74, 15.76)	0.11
Thyroid disease (not cancer, including Grave's disease)	302	1.25 (0.62, 2.50)	0.53
**Still Menstruating Variable**			
	301	0.17 (0.02, 1.26)	0.083
**Diagnosis of PMS Variable**			
	301	1.20 (0.49, 2.93)	0.68
**Currently Perimenopausal Variable**			
	294	0.53 (0.12, 2.36)	0.4
Hormone Levels Checked for Perimenopausal Variable			
	18	1.29 (0.07, 24.38)	0.87
**Have Experienced Menopause Variable**			
	298	9.09 (1.22, 67.83)	***0.031***
**Menses and PD Medication**			
Medication felt less effective	302	4.65 (1.13, 19.21)	***0.034***
More off/irregular frequency periods	302	3.08 (0.84, 11.29)	0.09

**Hormone Replacement Therapy Variable**			
	288	1.36 (0.73, 2.55)	0.33
**Birth Control Variable**			
	202	0.55 (0.07, 4.50)	0.57
**Experience of Breast Feeding**	296		
Yes		0.86 (0.40, 1.84)	0.7
No		1.26 (0.49, 3.23)	0.63
N/A		-reference-	
**PD Onset**	296		
While I was still having regular periods		-reference-	
While I was going through perimenopause		0.90 (0.30, 2.72)	0.85
One year or more after my last menstrual period		0.59 (0.29, 1.22)	0.16
**History of Surgeries**			
Total hysterectomy (removal of uterus, cervix, ovaries, Fallopian tubes, and surrounding structures)	302	1.59 (0.77, 3.30)	0.21
Oophorectomy (surgical removal of one/unilateral or both/bilateral ovaries)	302	0.58 (0.17, 2.00)	0.38
Mastectomy	302	5.93 (1.54, 22.85)	***0.01***
Partial hysterectomy (just uterus)	302	0.58 (0.19, 1.71)	0.32
**Types of Hormone Replacement Therapy**			
Estrogen	302	1.25 (0.54, 2.90)	0.61
Progesterone/Progestin	302	1.10 (0.23, 5.34)	0.9
Estrogen and progesterone combination	302	1.98 (0.92, 4.29)	0.082
**Types of Birth Control**			
Pill	302	0.89 (0.48, 1.64)	0.7
Ring	302	1.48 (0.29, 7.54)	0.64
Hormonal IUD	302	1.27 (0.40, 4.03)	0.68
Copper IUD	302	0.73 (0.27, 1.99)	0.54
Tubal ligation	302	1.11 (0.46, 2.69)	0.81
**Pregnancy Experiences**			

In vitro fertilization	302	0.33 (0.01, 7.42)	0.48
C-section	302	1.01 (0.49, 2.09)	0.98
Vaginal birth	302	1.13 (0.62, 2.07)	0.69
Having children prior to PD diagnosis	302	0.96 (0.50, 1.85)	0.91
No children	302	0.93 (0.45, 1.94)	0.86
**Difficulties in Pregnancy Experiences**			
Conceiving (fertility)	302	0.89 (0.37, 2.13)	0.79
Childbirth	302	1.16 (0.48, 2.81)	0.74
Pregnancy (reaching full birth)	302	1.06 (0.41, 2.72)	0.9
**Pregnancy-related Aliments**			
Eclampsia or pre-eclampsia	302	0.30 (0.04, 2.34)	0.25
Diabetes during pregnancy	302	0.73 (0.09, 6.16)	0.77
Viral infection	302	2.24 (0.40, 12.55)	0.36
Bacterial infection	302	2.63 (0.74, 9.30)	0.13
Depression/anxiety during pregnancy	302	5.78 (1.70, 19.69)	***0.005***
Postpartum Depression	302	2.11 (0.87, 5.14)	0.1
Flair up migraines	302	2.56 (0.97, 6.75)	0.057
**Hormone-related Disorders**			
Polycystic Ovarian Syndrome	302	1.91 (0.65, 5.67)	0.24
Hypothyroidism	302	1.00 (0.47, 2.13)	0.99
Hyperthyroidism	302	0.81 (0.23, 2.89)	0.75
Diabetes Type II	302	0.66 (0.15, 3.03)	0.6
Migraines (no aura)	302	1.69 (0.77, 3.73)	0.19

Variables in italics: use Firth method
Dummy variables for each response option
Reference was used to for univariate modeling and for the outcome of moderate/severe phenotype

Supplemental Table 1C. Part III: Univariate Logistic Regression Results

	Sample Size	OR (95% CI)	p-value
**Age**			
	141	1.01 (0.96, 1.06)	0.72
Hispanic			
	141	0.58 (0.02, 18.20)	0.76
**White**			
	141	0.70 (0.07, 7.03)	0.76
**Genetic Status**	139		
GBA		1.20 (0.17, 8.66)	0.86
LRRK2		2.50 (0.34, 18.33)	0.37
GBA	141	1.81 (0.52, 6.28)	0.35
LRRK2	141	4.09 (1.15, 14.60)	***0.03***
PRKN	141	2.20 (0.38, 12.69)	0.38
None		0.53 (0.10, 2.88)	0.47
Other		-reference-	
**Medications**			
Levodopa	141	0.68 (0.28, 1.69)	0.41
Entacapone	140	2.13 (0.19, 24.45)	0.54
Carbidopa, Levodopa, and Entacapone (Stalevo)	139	1.04 (0.21, 5.20)	0.96
Pramipexole	139	1.59 (0.52, 4.88)	0.42
Ropinirole	139	0.44 (0.05, 3.63)	0.45
Rotigotine	139	6.87 (1.09, 43.41)	***0.04***
Rasagiline	139	0.83 (0.29, 2.43)	0.74
Selegiline (oral, sublingual)	140	0.57 (0.02, 18.04)	0.75
Amantadine (liquid, infusion)	139	1.77 (0.51, 6.16)	0.37
**Current Comorbidities**			
Arrythmia/Atrial Fibrillation	141	0.51 (0.06, 4.26)	0.53
Arthritis	141	0.54 (0.23, 1.26)	0.15

B12 deficiency	141	4.64 (1.51, 14.23)	***0.007***
Cancer	141	1.06 (0.11, 9.87)	0.96
Depression	141	1.40 (0.57, 3.45)	0.46
Diabetes Mellitus (adult onset)	141	2.20 (0.38, 12.69)	0.38
Hearing Loss	141	0.84 (0.31, 2.28)	0.73
Hyper cholesterolemia (High Cholesterol)	141	0.90 (0.35, 2.33)	0.82
Hypertension (High Blood Pressure)	141	1.27 (0.45, 3.52)	0.65
Loss of smell	141	1.65 (0.71, 3.84)	0.24
Lung disease (including emphysema)	141	2.25 (0.53, 9.64)	0.27
Other	141	0.41 (0.15, 1.09)	0.073
Peripheral neuropathy	141	0.75 (0.16, 3.60)	0.72
PTSD	141	2.73 (0.61, 12.19)	0.19
Recreational drug use	141	0.59 (0.07, 4.99)	0.63
Renal insufficiency (kidney disease)	141	0.58 (0.02, 18.20)	0.76
Thyroid disease (not cancer, including Grave's disease)	141	0.65 (0.21, 2.07)	0.47
**Still Menstruating Variable**			
	141	1.06 (0.28, 4.06)	0.93
**Diagnosis of PMS Variable**			
	141	1.46 (0.37, 5.80)	0.59
Currently Perimenopausal Variable			
	137	0.18 (0.01, 3.69)	0.27
Hormone Levels Checked for Perimenopausal Variable			
**Have Experienced Menopause Variable**			
	138	0.70 (0.23, 2.12)	0.53
**Menses and PD Medication**			
Medication felt less effective	141	2.15 (0.19, 24.66)	0.54
More off/irregular frequency periods	141	1.06 (0.11, 9.87)	0.96
**Hormone Replacement Therapy Variable**			

	133	1.97 (0.81, 4.79)	0.13
Birth Control Variable			
	100	0.41 (0.02, 11.28)	0.6
**Experience of Breast Feeding**	140		
Yes		3.33 (0.73, 15.28)	0.12
No		4.81 (0.82, 28.27)	0.082
N/A		-reference-	
**PD Onset**	138		
While I was still having regular periods		-reference-	
While I was going through perimenopause		3.43 (0.79, 14.85)	0.1
One year or more after my last menstrual period		0.76 (0.25, 2.33)	0.63
**History of Surgeries**			
Total hysterectomy (removal of uterus, cervix, ovaries, Fallopian tubes, and surrounding structures)	141	3.30 (1.25, 8.68)	***0.016***
Oophorectomy (surgical removal of one/unilateral or both/bilateral ovaries)	141	0.36 (0.04, 2.92)	0.34
Mastectomy	141	1.06 (0.11, 9.87)	0.96
Partial hysterectomy (just uterus)	141	0.15 (0.01, 2.91)	0.21
**Types of Hormone Replacement Therapy**			
Estrogen	141	2.13 (0.67, 6.74)	0.2
Progesterone/Progestin	141	0.36 (0.02, 8.87)	0.53
Estrogen and progesterone combination	141	2.43 (0.82, 7.20)	0.11
**Types of Birth Control**			
Pill	141	1.11 (0.43, 2.89)	0.82
Ring	141	1.42 (0.14, 14.24)	0.76
Hormonal IUD	141	1.66 (0.41, 6.71)	0.48
Copper IUD	141	0.68 (0.14, 3.24)	0.63
Tubal ligation	141	0.97 (0.26, 3.68)	0.97
**Pregnancy Experiences**			
In vitro fertilization	141	0.45 (0.02, 12.03)	0.63

C-section	141	1.34 (0.48, 3.75)	0.57
Vaginal birth	141	1.04 (0.43, 2.53)	0.93
Having children prior to PD diagnosis	141	1.30 (0.45, 3.77)	0.63
No children	141	0.59 (0.16, 2.14)	0.42
**Difficulties in Pregnancy Experiences**			
Conceiving (fertility)	141	0.63 (0.17, 2.29)	0.48
Childbirth	141	0.46 (0.10, 2.11)	0.31
Pregnancy (reaching full birth)	141	1.50 (0.49, 4.56)	0.47
**Pregnancy-related Aliments**			
Eclampsia or pre-eclampsia	141	3.79 (0.94, 15.22)	0.06
Diabetes during pregnancy	141	1.42 (0.14, 14.24)	0.76
Bacterial infection	141	9.04 (0.79, 103.63)	0.077
Depression/anxiety during pregnancy	141	0.69 (0.08, 6.00)	0.74
Postpartum Depression	141	0.13 (0.01, 2.41)	0.17
Flair up migraines	141	0.93 (0.19, 4.59)	0.93
**Hormone-related Disorders**			
Polycystic Ovarian Syndrome	141	0.84 (0.09, 7.49)	0.87
Hypothyroidism	141	0.63 (0.17, 2.29)	0.48
Hyperthyroidism	141	0.20 (0.01, 4.17)	0.3
Diabetes Type II	141	2.20 (0.38, 12.69)	0.38
Migraines (no aura)	141	2.68 (0.89, 8.04)	0.079

Variables in italics: use Firth method
Dummy variables for each response option
Reference was used to for univariate modeling and for the outcome of moderate/severe phenotype

Supplemental Table 1D. Part IV: Univariate Logistic Regression Results

	Sample Size	OR (95% CI)	p-value
**Age**			
	139	1.01 (0.97, 1.05)	0.64
Hispanic			
	139	0.31 (0.01, 9.58)	0.5
**White**			
	139	2.31 (0.26, 20.37)	0.45
**Genetic Status**	138		
GBA		2.63 (0.40, 17.46)	0.32
LRRK2		3.00 (0.40, 22.71)	0.29
GBA	139	2.14 (0.72, 6.34)	0.17
LRRK2	139	2.39 (0.66, 8.75)	0.19
PRKN	139	1.12 (0.20, 6.37)	0.9
None		1.15 (0.22, 6.00)	0.87
Other		-reference-	
**Medications**			
Levodopa	139	1.42 (0.52, 3.88)	0.49
Entacapone	138	1.11 (0.10, 12.55)	0.93
Carbidopa, Levodopa, and Entacapone (Stalevo)	137	2.38 (0.72, 7.86)	0.16
Pramipexole	137	1.11 (0.41, 2.99)	0.83
Ropinirole	137	1.93 (0.56, 6.71)	0.3

Rotigotine	137	4.72 (0.83, 26.83)	0.08
Rasagiline	137	0.82 (0.34, 1.95)	0.65
Selegiline (oral, sublingual)	138	1.11 (0.10, 12.55)	0.93
Amantadine (liquid, infusion)	137	2.46 (0.85, 7.06)	0.095
**Current Comorbidities**			
Arrythmia/Atrial Fibrillation	139	0.89 (0.17, 4.77)	0.89
Arthritis	139	0.93 (0.45, 1.92)	0.85
B12 deficiency	139	2.91 (0.98, 8.62)	0.054
Cancer	139	0.43 (0.05, 3.83)	0.45
Depression	139	2.42 (1.12, 5.23)	***0.024***
Diabetes Mellitus (adult onset)	139	0.43 (0.05, 3.83)	0.45
Hearing Loss	139	1.02 (0.43, 2.39)	0.96
Hyper cholesterolemia (High Cholesterol)	139	0.79 (0.35, 1.79)	0.58
Hypertension (High Blood Pressure)	139	0.87 (0.35, 2.16)	0.76
Loss of smell	139	2.21 (1.07, 4.61)	***0.033***
Lung disease (including emphysema)	139	1.97 (0.57, 6.86)	0.28
Other	139	1.25 (0.59, 2.62)	0.56
Peripheral neuropathy	139	1.02 (0.33, 3.13)	0.98
PTSD	139	4.08 (0.93, 17.92)	0.063
Recreational drug use	139	0.62 (0.12, 3.12)	0.56

Renal insufficiency (kidney disease)	139	2.29 (0.31, 16.84)	0.41
Thyroid disease (not cancer, including Grave's disease)	139	0.95 (0.39, 2.28)	0.9
**Still Menstruating Variable**			
	139	0.18 (0.02, 1.47)	0.11
**Diagnosis of PMS Variable**			
	139	2.43 (0.74, 8.03)	0.14
Currently Perimenopausal Variable			
	136	0.62 (0.12, 3.13)	0.56
Hormone Levels Checked for Perimenopausal Variable			
	9	0.75 (0.03, 17.51)	0.86
**Have Experienced Menopause Variable**			
	136	1.52 (0.47, 4.99)	0.49
**Menses and PD Medication**			
Medication felt less effective	139	9.74 (1.06, 89.96)	***0.045***
More off/irregular frequency periods	139	4.82 (0.85, 27.41)	0.076
**Hormone Replacement Therapy Variable**	132	0.57 (0.24, 1.35)	0.2
**Birth Control Variable**			
	96	0.69 (0.07, 6.90)	0.75
**Experience of Breast Feeding**	138		
Yes		1.36 (0.49, 3.78)	0.55
No		2.59 (0.73, 9.25)	0.14
N/A		-reference-	

**PD Onset**	136		
While I was still having regular periods		-reference-	
While I was going through perimenopause		1.94 (0.50, 7.64)	0.34
One year or more after my last menstrual period		0.63 (0.25, 1.60)	0.33
**History of Surgeries**			
Total hysterectomy (removal of uterus, cervix, ovaries, Fallopian tubes, and surrounding structures)	139	0.97 (0.37, 2.57)	0.95
Oophorectomy (surgical removal of one/unilateral or both/bilateral ovaries)	139	2.43 (0.74, 8.03)	0.14
Mastectomy	139	3.52 (0.57, 21.91)	0.18
Partial hysterectomy (just uterus)	139	0.99 (0.29, 3.42)	0.99
**Types of Hormone Replacement Therapy**			
Estrogen	139	1.02 (0.33, 3.13)	0.98
Progesterone/Progestin	139	0.19 (0.01, 4.66)	0.31
Estrogen and progesterone combination	139	0.84 (0.28, 2.53)	0.76
**Types of Birth Control**			
Pill	139	1.17 (0.53, 2.60)	0.69
Ring	139	0.24 (0.01, 6.32)	0.39
Hormonal IUD	139	0.83 (0.21, 3.27)	0.78
Copper IUD	139	2.47 (0.81, 7.55)	0.11
Tubal ligation	139	1.77 (0.65, 4.76)	0.26
**Pregnancy Experiences**			
In vitro fertilization	139	1.12 (0.10, 12.68)	0.93
C-section	139	0.66 (0.24, 1.78)	0.41

Vaginal birth	139	1.35 (0.62, 2.98)	0.45
Having children prior to PD diagnosis	139	2.17 (0.82, 5.76)	0.12
No children	139	0.75 (0.27, 2.07)	0.58
**Difficulties in Pregnancy Experiences**			
Conceiving (fertility)	139	1.04 (0.37, 2.94)	0.95
Childbirth	139	1.04 (0.37, 2.94)	0.95
Pregnancy (reaching full birth)	139	1.60 (0.60, 4.25)	0.35
**Pregnancy-related Aliments**			
Eclampsia or pre-eclampsia	139	3.18 (0.68, 14.87)	0.14
Diabetes during pregnancy	139	9.74 (1.06, 89.96)	***0.045***
Viral infection	139	6.72 (0.07, 620.49)	0.41
Bacterial infection	139	12.49 (1.41, 110.45)	***0.023***
Depression/anxiety during pregnancy	139	1.73 (0.37, 8.07)	0.49
Postpartum Depression	139	0.58 (0.15, 2.19)	0.42
Flair up migraines	139	5.03 (1.19, 21.16)	***0.028***
**Hormone-related Disorders**			
Polycystic Ovarian Syndrome	139	1.73 (0.37, 8.07)	0.49
Hypothyroidism	139	0.90 (0.34, 2.37)	0.84
Hyperthyroidism	139	1.13 (0.27, 4.72)	0.87
Diabetes Type II	139	0.16 (0.01, 3.65)	0.25
Migraines (no aura)	139	0.84 (0.28, 2.53)	0.76

Variables in italics: use Firth method
Dummy variables for each response option
Reference was used to for univariate modeling and for the outcome of moderate/severe phenotype

Supplemental Table 2: List of Comorbidities

Variable	Label	Type	Length	Codes	Format name
SUBJCAP		Char	8		
COMORBID	Have you ever been diagnosed with or treated for any of these conditions?	Char	12	1 = a) Hypertension (High Blood Pressure)	COMORBID
				2 = b) Diabetes Mellitus (childhood onset)	COMORBID
				3 = c) Diabetes Mellitus (adult onset)	COMORBID
				4 = d) Myocardial Infarction (Heart Attack)	COMORBID
				5 = e) Congestive Heart Failure	COMORBID
				6 = f) Arrythmia/Atrial Fibrillation	COMORBID
				7 = g) Hyper cholesterolemia (High Cholesterol)	COMORBID
				8 = h) Hearing Loss	COMORBID
				9 = i) Loss of smell	COMORBID
				10 = j) Lung disease (including emphysema)	COMORBID
				11 = k) Thyroid disease (not cancer; including Grave's disease)	COMORBID
				12 = l) Liver	COMORBID
				13 = m) Renal insufficiency (kidney disease)	COMORBID
				14 = n) Peptic ulcer disease	COMORBID
				15 = o) Peripheral vascular disease	COMORBID
				16 = p) Stroke, mini stroke, CVA (cerebrovascular accident) or TIA (transient ischemic attack)	COMORBID
				17 = q) Seizure, fit , convulsion or unexplained loss of consciousness	COMORBID
				18 = r) Arthritis	COMORBID
				19 = s) Hip Fracture	COMORBID
				20 = t) Gout	COMORBID
				21 = u) Recreational drug use	COMORBID
				22 = v) Gaucher disease	COMORBID
				23 = w) Multiple sclerosis	COMORBID
				24 = x) Alzheimer's disease	COMORBID
				25 = y) ALS	COMORBID
				26 = z) PTSD	COMORBID
				27 = aa) Depression	COMORBID
				28 = bb) Normal pressure hydrocephalus	COMORBID
				29 = cc) Peripheral neuropathy	COMORBID
				30 = dd) Crohn's disease/ulcerative colitis	COMORBID
				31 = ee) B12 deficiency	COMORBID
				32 = ff) Cancer, if yes please specify:	COMORBID

Supplemental Table 2: List of Comorbidities

Variable	Label	Type	Length	Codes	Format name
				33 = gg) Other, if yes please specify:	COMORBID
CURRENT	Current Diagnosis	Num	8	0 = No	YN
				1 = Yes	YN
LIFETIME	Lifetime Diagnosis	Num	8	0 = No	YN
				1 = Yes	YN
CANCERNM	Cancer, specify	Char	51		
OTHERNM	Other Medical Problems	Char	40		
C_BRAIN	Did you ever have a brain surgery?	Num	8	0 = No	YNUNK
				1 = Yes	YNUNK
				9 = Unknown	YNUNK

Supplemental Table 3. Frequency of UPDRS Scores, by Severity Groups Parts I-IV

	Part I	Part II	Part III	Part IV
UPDRS Moderate/Severe Threshold	>21	>29	>58	>12
Mild n (%)	112 (70.0)	246 (81.46)	114 (80.85)	96 (69.06)
Moderate/Severe n (%)	48 (30.0)	56 (18.54)	27 (19.15)	40 (30.94)
