# Supplementary material for: Association of women-specific health factors in the severity of Parkinson’s disease
Source: NPJ Parkinsons Dis. 2023 Jun 5;9:86. doi: 10.1038/s41531-023-00524-x (PMC10241917; doi:10.1038/s41531-023-00524-x)
Supplement: Supplementary file 2 — Reporting Summary [file 41531_2023_524_MOESM2_ESM.pdf]

nature portfolio

Corresponding author(s): Ignacio Mata

Last updated by author(s): May 5, 2023

Reporting Summary

Nature Portfolio wishes to improve the reproducibility of the work that we publish. This form provides structure for consistency and transparency in reporting. For further information on Nature Portfolio policies, see our Editorial Policies and the Editorial Policy Checklist.

Statistics

For all statistical analyses, confirm that the following items are present in the figure legend, table legend, main text, or Methods section.

n/a	Confirmed	
☐	☒	The exact sample size (*n*) for each experimental group/condition, given as a discrete number and unit of measurement
☐	☒	A statement on whether measurements were taken from distinct samples or whether the same sample was measured repeatedly
☐	☒	The statistical test(s) used AND whether they are one- or two-sided<br>*Only common tests should be described solely by name; describe more complex techniques in the Methods section.*
☐	☒	A description of all covariates tested
☐	☒	A description of any assumptions or corrections, such as tests of normality and adjustment for multiple comparisons
☐	☒	A full description of the statistical parameters including central tendency (e.g. means) or other basic estimates (e.g. regression coefficient) AND variation (e.g. standard deviation) or associated estimates of uncertainty (e.g. confidence intervals)
☐	☒	For null hypothesis testing, the test statistic (e.g. *F*, *t*, *r*) with confidence intervals, effect sizes, degrees of freedom and *P* value noted<br>*Give P values as exact values whenever suitable.*
☒	☐	For Bayesian analysis, information on the choice of priors and Markov chain Monte Carlo settings
☒	☐	For hierarchical and complex designs, identification of the appropriate level for tests and full reporting of outcomes
☒	☐	Estimates of effect sizes (e.g. Cohen's *d*, Pearson's *r*), indicating how they were calculated

Our web collection on statistics for biologists contains articles on many of the points above.

Software and code

Policy information about availability of computer code

Data collection	The questionnaire, was created and distributed via REDCap, a secure web application used for building online surveys and databases.
Data analysis	All statistical analyses were conducted using SAS version 9.4 (SAS Institute Inc, Cary, NC). Statistical significance was established throughout at p<0.05

For manuscripts utilizing custom algorithms or software that are central to the research but not yet described in published literature, software must be made available to editors and reviewers. We strongly encourage code deposition in a community repository (e.g. GitHub). See the Nature Portfolio guidelines for submitting code & software for further information.

Data

Policy information about availability of data

All manuscripts must include a data availability statement. This statement should provide the following information, where applicable:

- Accession codes, unique identifiers, or web links for publicly available datasets
- A description of any restrictions on data availability
- For clinical datasets or third party data, please ensure that the statement adheres to our policy

The datasets generated during and/or analyzed during the current study are available from the corresponding author upon request. Please submit a request to the Parkinson’s Foundation PD GENEration by email to srao@parkinson.org.

Research involving human participants, their data, or biological material

Policy information about studies with human participants or human data. See also policy information about sex, gender (identity/presentation), and sexual orientation and race, ethnicity and racism.

Reporting on sex and gender	As this is study focuses on women-specific health factors, there is no sex/ gender differences analyses. This study aims to highlight experiences pertaining to those that experience women-specific factors ranging from menses to menopause.
Reporting on race, ethnicity, or other socially relevant groupings	We utilized self-reported ethnicity (not Hispanic/Latino or Hispanic/Latino) present in the PD GENEration questionnaire. For race we used categories such as "White", "Black", "Asian", "American Indian/ Alaska Native", "Native Hawaiian/Pacific Islander", and "Other".
Population characteristics	The questionnaire was sent to 966 women with PD enrolled with the Parkinson's Foundation PD GENEration. Of the returned surveys, 304 women gave complete responses, providing a response rate of 31.5%. Overall, respondents averaged 64.7 years in age (±9.1), with a mean age at diagnosis of 58.5 years (±10.4). Women completing the survey had an average of 6.2 years of disease duration (±5.3) since they were diagnosed with PD. Within our cohort, we have women with GBA (10%), LRRK2 (8%), PINK1 (1%), PRKN (4%), and VPS35 (1.3%) mutations.
Recruitment	PD GENEration released our questionnaire cross-sectionally to women with PD who had participated in PD GENEration in order to retrospectively inquire about their women-specific experiences and how these experiences may have impacted their PD health-related quality of life.
Ethics oversight	Cleveland Clinic, The Parkinson's Foundation

Note that full information on the approval of the study protocol must also be provided in the manuscript.

Field-specific reporting

Please select the one below that is the best fit for your research. If you are not sure, read the appropriate sections before making your selection.

☐ Life sciences ☒ Behavioural & social sciences ☐ Ecological, evolutionary & environmental sciences

For a reference copy of the document with all sections, see nature.com/documents/nr-reporting-summary-flat.pdf

Behavioural & social sciences study design

All studies must disclose on these points even when the disclosure is negative.

Study description	We sought to identify women-specific health experiences associated with PD severity, after adjusting for known PD factors, by developing and distributing a women-specific questionnaire across the United States and creating multivariable models for PD severity. We created a questionnaire that addresses women's specific experiences and their PD clinical history and deployed it through The Parkinson's Foundation: PD Generation. To determine the association between women-specific health factors and PD severity, we constructed multivariable logistic regression models based on the MDS-UPDRS scale and the participants' questionnaire responses, genetics, and clinical data.
Research sample	Research sample was provided on behalf of PD GENEration. With all respondents being self-reported as women (sex assigned at birth was also female) and with an average age of 64.7 (±9.1), with a mean age at diagnosis of 58.5 years (±10.4). Women completing the survey had an average of 6.2 years of disease duration (±5.3) since they were diagnosed with PD. All participants are within the United States.
Sampling strategy	PD GENEration released our questionnaire cross-sectionally to women with PD who had participated in PD GENEration retrospectively. Power analysis demonstrated that our cohort sizes were sufficient for a proper analysis. This was a non-probability sampling.
Data collection	Clinical history and demographics were collected by PD GENEration through REDCap. The women-specific questionnaire was also done through REDCap.
Timing	Starting in mid-November 2002, PD GENEration released our questionnaire cross-sectionally to women with PD who had participated in PD GENEration in order to retrospectively inquire about their women-specific experiences and how these experiences may have impacted their PD health-related quality of life. An email reminder was sent in mid-December, and the questionnaire closed on December 2021.
Data exclusions	As this is a patient-reported study, we filtered the variables in the questionnaire and demographic data from PD generation, and we removed variables where at least 80% of responses were missing or "not available".
Non-participation	The questionnaire was sent to 966 women with PD enrolled with the Parkinson's Foundation PD GENEration. Of the returned surveys, 304 women gave complete responses, providing a response rate of 31.5%.
Randomization	Participants were divided our cohort into "mild" and "moderate or severe" UPDRS groups based on the maximum triangulation cut-off values stated in a previous study conducted by Martinez-Martin et al. We decided to group them by "mild" vs. "moderate/severe"

due to the low number of women with a "severe" UPDRS score. Thresholds for moderate/severe were Part I: >21; Part II: >29; Part III: >58; and Part IV: >12.

Reporting for specific materials, systems and methods

We require information from authors about some types of materials, experimental systems and methods used in many studies. Here, indicate whether each material, system or method listed is relevant to your study. If you are not sure if a list item applies to your research, read the appropriate section before selecting a response.

Materials & experimental systems

n/a	Involved in the study
☒	☐ Antibodies
☒	☐ Eukaryotic cell lines
☒	☐ Palaeontology and archaeology
☒	☐ Animals and other organisms
☐	☒ Clinical data
☒	☐ Dual use research of concern
☒	☐ Plants

Methods

n/a	Involved in the study
☒	☐ ChIP-seq
☒	☐ Flow cytometry
☒	☐ MRI-based neuroimaging

Clinical data

Policy information about clinical studies

All manuscripts should comply with the ICMJE guidelines for publication of clinical research and a completed CONSORT checklist must be included with all submissions.

Clinical trial registration

NCT04057794

Study protocol

https://www.parkinson.org/sites/default/files/documents/PDGENEration-Protocol.pdf

Data collection

Starting in mid-November 2002, PD GENEration released our questionnaire cross-sectionally to women with PD who had participated in PD GENEration in order to retrospectively inquire about their women-specific experiences and how these experiences may have impacted their PD health-related quality of life. An email reminder was sent in mid-December, and the questionnaire closed on December 2021. All participants provided written consent to be part of this study.

Outcomes

Participants were divided our cohort into "mild" and "moderate or severe" UPDRS groups based on the maximum triangulation cut-off values stated in a previous study conducted by Martinez-Martin et al. We decided to group them by "mild" vs. "moderate/severe" due to the low number of women with a "severe" UPDRS score. Thresholds for moderate/severe were Part I: >21; Part II: >29; Part III: >58; and Part IV: >12.
